# Effect of different citrus sweets on the development of enamel erosion *in vitro*

**DOI:** 10.1590/1678-7757-2020-0182

**Published:** 2020-08-17

**Authors:** Beatriz Martines de SOUZA, Mariele VERTUAN, Isabela Vieira Bolzan GONÇALVES, Ana Carolina MAGALHÃES

**Affiliations:** 1 Laboratório de Bioquímica Faculdade de Odontologia de Bauru Universidade de São Paulo BauruSão Paulo Brasil Departamento de Ciências Biológicas, Laboratório de Bioquímica , Faculdade de Odontologia de Bauru da Universidade de São Paulo , Bauru , São Paulo , Brasil .

**Keywords:** Dental enamel, Sweet, Tooth erosion, Tooth wear

## Abstract

The increased consumption of citrus sweets can contribute to the development of erosive tooth wear (ETW).

**Objective:**

This in vitro study evaluated the erosive potential of citrus sweets on bovine enamel samples regarding the quantification of wear.

**Methodology:**

Ninety bovine crowns were prepared and samples were randomly distributed into 6 groups (n=15): 0.1% citric acid solution (pH 2.5); Coca-Cola ^®^ Soft Drink (pH 2.6); Fini ^®^ Diet (lactic and citric acid, pH 3.3); Fini ^®^ Jelly Kisses (lactic and citric acid, pH 3.5); Fini ^®^ Fruit Salad Bubblegum (maleic acid, pH 2.6); Fini ^®^ Regaliz Acid Tubes (maleic and citric acid, pH 3.1). Sweets were dissolved in the proportion of 40 g/250 mL of deionized water. Enamel samples were submitted to erosive challenges for 7 days (4 daily acid immersion cycles for 90 s each). Enamel wear was measured using contact profilometry (μm), and data (median values [interquartile range]) were submitted to Kruskal-Wallis/Dunn’s test (p<0.0001).

**Results:**

All citrus sweets tested present a high erosive potential, Fini Diet ^®^ (2.4 [1.2]) and Fini Regaliz Tubs ^®^ (2.2 [0.5]) show the highest erosive potential, similar to 0.1% citric acid (2.3 [0.7]); Fini Regaliz Tubs ^®^ is more erosive than Coca-Cola ^®^ (1.4 [0.9]).

**Conclusion:**

The evaluated citrus sweets have great erosive potential and play a key role in the development of ETW.

## Introduction

Tooth erosion is a well-known condition of tooth wear caused by exposure to acid (erosive tooth wear - ETW) that has a multifactorial etiology, involving chemical, biological, and behavioral factors. ^1^ It starts as tooth softening and further progresses into wear, due to the cumulative effects of erosive challenges associated with mechanical forces such as abrasion and attrition. ^2^ Sensitivity to pain, poor functioning and aesthetics, and inflammation of the dental pulp are examples of consequences of ETW. ^2^

Interest in this topic has grown exponentially among researchers and clinicians ^3^ due to its increasing prevalence, especially among children and young people, and its severity, which tends to increase with increasing age. ^4 - 6^ Tschammler, et al. ^6^ (2016) showed that the prevalence of tooth erosion increased from 25% to 50% in 10 years among 3-6-year-old German children. ^6^ This finding corroborates systematic reviews that have shown a prevalence of such condition of about 50% in deciduous dentition, and of 30% in permanent dentition. ^5 , 7 , 8^ The most alarming finding is that erosion in deciduous dentition increases about four times the chance its development in permanent dentition; ^9^ and, even in permanent dentition, the likelihood for erosion progression is higher among individuals with previous experience than among those who have never had this condition. ^10^

Acid exposure may occur due to intrinsic sources (gastric hydrochloric acid) and/or extrinsic (food and beverages containing citric, phosphoric, and/or maleic acids). ^1 , 11^ Dietary habits involving frequent intake of acidic food and beverage between meals may increase the risk of developing ETW. ^5 , 12 - 14^

Although soft drinks are considered the villains for tooth erosion development, an important systematic review showed that the odds ratio for the development of the condition is higher for individuals who often consume citrus sweets (2.24 x) than for those who consume soft drink (1.61 x). ^5^ Such finding evinces the clinical impact of daily consumption of citrus sweets on tooth erosion development – not only because they are acidic when diluted in saliva, but also because they adhere to the tooth surface, prolonging the deleterious effect. ^15^

Sweets are often reported as a cariogenic food, but not as an erosive agent. Despite the aforementioned systematic review, ^5^ few studies have been made for proof-of-concept and/or comparing different sweets for their erosive potential. Recently, Carvalho, et al. ^16^ (2017) evaluated the erosive potential of different foods, including a citrus sweet brand (Haribo Gold Bears Gummi Candy, apple flavor, containing maleic, citric, and tartaric acid), showing its potential to cause enamel softening in both deciduous and permanent dentition after 2 and 4 minutes of exposure. ^16^ Likewise, Shen, et al. ^17^ (2017) demonstrated the great erosive potential of 17 out of 30 bullet brands (no sugar), which was assessed by loss of enamel microhardness. ^17^

Considering that children and young people are the main consumers of citrus sweets and that the prevalence of tooth erosion is significant at this stage of life, our study aimed to evaluate the effect of citrus sweets (with and without sugar) on the development of erosive enamel wear, by comparing these sweets to known erosive agents (such as soft drink) *in vitro* . Our null hypothesis was that there is no significant difference between the tested sweets and soft drink on the development of erosive enamel wear.

## Methodology

This study followed the CRIS guidelines for in-vitro studies, as described in the 2014 concept note. ^18^

### Sample preparation

This study was approved by the Local Ethics Committee on Animal Experimentation (No. 06/2019). Ninety enamel samples from recently extracted bovine incisors were kept in 0.1% thymol solution (pH 7.0) and then prepared. Root and crown were separated using a cutting machine (Maruto, Tokyo, Japan) and a diamond disc (Maruto, Tokyo, Japan). The crowns were coupled to a premade silicone mold (Biopdi, São Carlos, Brazil) and embedded into autopolymerizing acrylic resin, enabling buccal surface exposure. The samples were polished using silicon carbide sandpapers (320, 600, and 1,200-grit papers of Al _2_ O _3_ ; Extec Corp., USA) in a polishing machine. When sandpapers were changed and at the end of polishing, samples were ultrasonic rinsed in deionized water (Ultrasonic-T14, L&R Ultrasonic, USA) for 2 minutes.

Baseline profile was measured using a contact profilometer (Mahr, Germany). To guarantee repeatability in measures, the samples had identification marks: two scalpel scratches, which allowed the delimitation of control areas, and a small drilling made with a ¼-inch drill (Jet Carbide, Kerr, Joinvile, Brazil), to standardize where reading began. Samples were inserted in a metal device able to reproduce the reading position based on their location within the x and y axes, ^19^ allowing the stylus to be accurately repositioned at each measurement (baseline and final).

**Figure 1:**
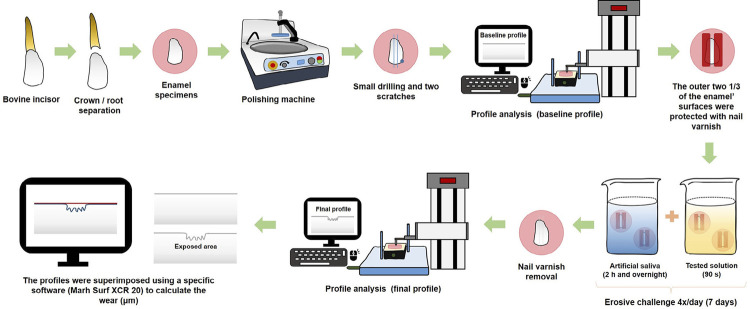
Study design showing the experimental steps

After baseline profile, the outer two 1/3 of the enamel surface was protected with nail varnish (Risqué, São Paulo, Brazil) to obtain 2 control areas – indispensable for tooth wear quantification. This nail varnish remained on the samples throughout the erosive challenges, protecting the control surfaces (sound surfaces). Figure 1 shows the study design described below.

### Experimental groups

Ninety enamel samples were randomly divided into 6 groups (n=15): Group 1 – 0.1% citric acid solution (standard control, pH 2.5); Group 2 – Coca-Cola Soft Drink (Coca-Cola ^®^ , phosphoric acid, MW: 97.994 g/mol, pH 2.6 - expiry date: 07/09/19, lot: P240619); Group 3 – Fini ^®^ Diet (without sugar; lactic acid MW: 90.08 g/mol and citric acid MW: 192.123 g/mol, pH 3.3 - expiry date: 01/2020, lot: GNSD804); Group 4 – Fini ^®^ Jelly Kisses, strawberry flavor (citric acid and lactic acid, pH 3.5 - expiry date: 01/2020, lot: GBMR831); Group 5 – Fini ^®^ Fruit Salad Bubblegum (maleic acid, MW 116.1 g/mol, pH 2.6 - expiry date: 08/2020, lot: CSAL835); Group 6 – Fini ^®^ Regaliz Acid Tubes, tutti-frutti flavor (maleic acid and citric acid, pH 3.1 - expiry date: 08/2020, lot: RTMC838L2). The citrus sweets were selected considering their availability in the city’s main supermarkets. Solutions were prepared by mixing 40 g of sweets in 250 ml of deionized water, at room temperature, until complete dissolution. ^20^

### Erosive challenges

Samples were submitted to erosive challenges during 7 days, ^21 - 23^ four times a day, as follows: (1) demineralization by immersion in tested-sweet solutions for 90 s (30 mL/sample), at 25°C, without stirring; (2) rinse in deionized water (5 s); (3) remineralization by immersion in artificial saliva for 2 h (pH 6.8, 30 mL/sample), at 25°C, without stirring; (4) rinse in deionized water again (5 s).

The artificial saliva was composed of the following reagents: 0.2 mM glucose, 9.9 mM NaCl, 1.5 mM CaCl _2_ **∙** 2H _2_ O, 3 mM NH _4_ Cl, 17 mM KCl, 2 mM NaSCN, 2.4 mM K _2_ HPO _4_ , 3.3 mM urea, 2.4 mM NaH _2_ PO _4,_ and traces of ascorbic acid (pH 6.8). ^24^ Samples were kept in artificial saliva overnight, completing 24 h of cycling. Acid solutions were renewed at each challenge and artificial saliva once a day (before the first erosive challenge of the day). After 7 days of erosive challenges, the final surface profile was measured to quantify enamel wear.

### Contact profilometry

Surface profiles were obtained using a contact profilometer (Mahr, Göttingen, Germany), at baseline and after 7 days of pH cycling. Five equidistant surface scans were performed for each enamel sample (6 mm of reading, 250 μm apart from each other) at midpoint surface, in the mesiodistal direction of the crown. Samples were positioned in a metal device, which has been previously described, ^22 - 23^ enabling the standardization of samples position at each measurement (baseline and final).

To determine enamel wear after erosive challenges, the nail varnish was removed using an acetone solution (1:1 - acetone: water) to avoid any interference on the measurement. Then, 5 readings were performed at the same areas of the baseline readings. For calculating dental wear, baseline profiles were superposed to final profiles using a specific software (Marh Surf XCR 20) and average height differences were calculated (μm), considering 0.1 μm as the limit of detection. ^19^ The average enamel wear was calculated using the five readings of each sample and then used to compare the erosive potential of different citric sweets in relation to the controls (soft drink and citric acid).

### Statistical analysis

The average erosive enamel wear (μm) of each sample was tabulated in Excel. Graph Pad Prism (USA) was used for the statistical analysis. The data passed the normality test (Komogorov-Smirnov test), but they were not homogeneous (Bartlett test). Data were compared using Kruskal-Wallis test followed by post-hoc Dunn’s test. Significance level was set at 5%.

## Results

All citric sweets evaluated induced enamel wear at different degrees. Among them, Fini ^®^ Diet and Fini ^®^ Regaliz Tubs showed the highest erosive potential – similar to 0.1% citric acid. Erosion was significantly greater with Fini ^®^ Regaliz Tubs than with Coca-Cola ^®^ . Fini ^®^ Kisses and Fini ^®^ Fruit Salad caused the lowest erosive wear, which, although comparable to Coca-Cola ^®^ , was significantly lower than Fini ^®^ Diet and Fini ^®^ Regaliz Tubs ( Table 1 ).

**Table 1:** Median values (interquartile range, II) of erosive enamel wear (µm) according to each acid solution

Treatments	Median (II)
0.1% Citric acid	2.3 (0.7) ^a^
Coca-Cola ^®^	1.4 (0.5) ^bc^
Fini ^®^ Diet	2.4 (1.2) ^ab^
Fini ^®^ Kisses	1.4 (0.9) ^c^
Fini ^®^ Bubblegum Fruit Salad	1.3 (0.3) ^c^
Fini ^®^ Regaliz Acid Tubes	2.2 (0.5) ^a^

Different lower-case letters point out significant differences among the groups (Kruskal-Wallis/Dunn's test, p<0.0001, n=15).

## Discussion

This study null hypothesis was that there would be no significant difference between the tested sweets and soft drink on the development of erosive enamel wear. However, our results refuted it, as one of the sweets was more erosive than Coca-Cola ^®^ (phosphoric acid), but comparable to the control (0.1% citric acid). The evaluated sweets contained maleic, citric, and/or lactic acids, but their label did not describe acids concentrations. Our results suggest that sweets containing citric acid combined with other acid (lactic or maleic) tend to be more erosive than those containing only maleic acid, regardless of the pH values. Besides pH values, other factors may determine foods/beverages erosive potential, such as acid type, acid concentration, titratable acidity, buffering capacity, molecular weight (influencing acid diffusion), and dissociated acid concentration. ^25^

Citric acid is highly aggressive not only because it releases H ^+^ at different steps of its cycle (pK _a_ -values: 3.1, 4.74, and 6.42), but also because it entails an additional effect caused by citrate ion, a calcium chelator, whose effect is more significant at pH > 4. ^26 - 28^ It is also considered a complex chemical compound, hampering the prediction of its erosive potential at different concentrations. ^25^ In our study, erosive effect was not associated with its chelating action, but rather with the high H ^+^ release, which was probably even more aggressive in the presence of other acid.

Our results are in line with those found in the systematic review, which indicates that acidic sweets have a higher impact on tooth erosion than soft drinks. ^5^ Different from soft drinks, sweets are viscous and can adhere to the tooth surface, enhancing their deleterious effect. ^15^ Unfortunately, our experimental model did not allow to test the effect of the adhesion of sweet on the tooth surface (caused by chewing) on the development of erosive enamel wear. However, if the model allowed us to test this property, we could expect an even higher enamel wear induced by sweets.

Considering that consuming acidic food and beverage between meals increases the chances of developing tooth erosion, ^5 , 29^ information on sweets erosive potential must be spread across the population, particularly for raising parents’ awareness regarding offering their children sweets, especially in between main meals.

Studies evaluating the erosive potential of citric sweets are scarce. The two *in vitro* studies mentioned throughtout this article, evaluated the impact of sweets on enamel softening (early stage of tooth erosion), but not on enamel wear. Carvalho, et al. ^16^ (2017) showed that a sweet (containing maleic, citric, and tartaric acid) presented the lowest pH value among different foods, and yet caused an enamel softening 6 times greater than an orange juice. Shen, et al. ^17^ (2017) tested the erosive potential of 30 sugar-free sweets and found that, from 19 acidic sweets, 17 induced enamel softening, especially those containing citric acid, corroborating our results. In our study, we found that the most erosive sweet was a diet one; however, even the sweets that contained sugar (considered cariogenic) caused erosive enamel wear.

## Conclusion

Citrus sweets cause erosive enamel wear at different degrees. Our results raise a red flag about the consumption of this type of sweets – especially because its main consumers are children and teenagers, who present a high prevalence of this condition. ^5 , 6 , 8 , 30^

## References

[1] - Kanzow P, Wegehaupt FJ, Attin T, Wiegand A. Etiology and pathogenesis of dental erosion. Quintessence Int. 2016;47(4):275-8. doi: 10.3290/j.qi.a35625 10.3290/j.qi.a35625 27022647

[2] - Passos VF, Melo MA, Park J, Strassler HE. Current concepts and best evidence on strategies to prevent dental erosion. Compend Contin Educ Dent. 2019;40(2):80-7. 30767547

[3] - Lussi A, Carvalho TS. Erosive tooth wear: a multifactorial condition of growing concern and increasing knowledge. Monogr Oral Sci. 2014;25:1-15. doi: 10.1159/000360380 10.1159/000360380 24993253

[4] - Aguiar YP, Santos FG, Moura EF, Costa FC, Auad SM, Paiva SM, et al. Association between dental erosion and diet in Brazilian adolescents aged from 15 to 19: a population-based study. Sci World J. 2014;818167. doi: 10.1155/2014/818167 10.1155/2014/818167 PMC3947812 24695943

[5] - Salas MM, Nascimento GG, Vargas-Ferreira F, Tarquinio SB, Huysmans MC, Demarco FF. Diet influenced tooth erosion prevalence in children and adolescents: results of a meta-analysis and meta-regression. J Dent. 2015;43(8):865-75. 10.1016/j.jdent.2015.05.012 10.1016/j.jdent.2015.05.012 26057086

[6] - Tschammler C, Müller-Pflanz C, Attin T, Müller J, Wiegand A. Prevalence and risk factors of erosive tooth wear in 3-6 year old German kindergarten children: a comparison between 2004/05 and 2014/15. J Dent. 2016;52:45-9. 10.1016/j.jdent.2016.07.003 10.1016/j.jdent.2016.07.003 27396612

[7] - Kreulen CM, Van't Spijker A, Rodriguez JM, Bronkhorst EM, Creugers NH, Bartlett DW. Systematic review of the prevalence of tooth wear in children and adolescents. Caries Res. 2010;44(2):151-9. doi: 10.1159/000308567 10.1159/000308567 20389070

[8] - Salas MM, Vargas-Ferreira F, Ardenghi TM, Peres KG, Huysmans MD, Demarco FF. Prevalence and associated factors of tooth erosion in 8 -12-year-old brazilian schoolchildren. J Clin Pediatr Dent. 2017;41(5):343-50. doi: 10.17796/1053-4628-41.5.34 10.17796/1053-4628-41.5.343 28872983

[9] - Ganss C, Klimek J, Giese K. Dental erosion in children and adolescents--a cross-sectional and longitudinal investigation using study models. Community Dent Oral Epidemiol. 2001;29(4):264-71. 10.1034/j.1600-0528.2001.290405.x 11515640

[10] - Brusius CD, Alves LS, Susin C, Maltz M. Dental erosion among South Brazilian adolescents: a 2.5-year longitudinal study. Community Dent Oral Epidemiol. 2018;46(1):17-23. doi: 10.1111/cdoe.12322 10.1111/cdoe.12322 28727163

[11] - Lussi A. Erosive tooth wear - a multifactorial condition of growing concern and increasing knowledge. Monogr Oral Sci. 2006;20:1-8. doi: 10.1159/000360380 10.1159/000093343 16687880

[12] - Lussi A, Carvalho TS. Analyses of the erosive effect of dietary substances and medications on deciduous teeth. PLoS One. 2015;10(12):e0143957. doi: 10.1371/journal.pone.0143957 10.1371/journal.pone.0143957 PMC4689448 26700481

[13] 13 - Gravelle BL, Hagen TW II, Mayhew SL, Crumpton B, Sanders T, Horne V. Soft drinks and *in vitro* dental erosion. Gen Dent 2015;63(4):33-8. 26147165

[14] - O'Toole S, Mullan F. The role of the diet in tooth wear. Br Dent J. 2018;224(5):379-83. doi: 10.1038/sj.bdj.2018.127 10.1038/sj.bdj.2018.127 29471309

[15] - Nadimi H, Wesamaa H, Janket SJ, Bollu P, Meurman JH. Are sugar-free confections really beneficial for dental health? Br Dent J. 2011;211(7):E15. doi: 10.1038/sj.bdj.2011.823 10.1038/sj.bdj.2011.823 21979369

[16] - Carvalho TS, Schmid TM, Baumann T, Lussi A. Erosive effect of different dietary substances on deciduous and permanent teeth. Clin Oral Investig 2017;21(5):1519-26. doi: 10.1007/s00784-016-1915-z 10.1007/s00784-016-1915-z 27449386

[17] - Shen P, Walker GD, Yuan Y, Reynolds C, Stacey MA, Reynolds EC. Food acid content and erosive potential of sugar-free confections. Aust Dent J. 2017;62(2):215-22. doi: 10.1111/adj.12498 10.1111/adj.12498 28107545

[18] 18 - Krithikadatta J, Gopikrishna V, Datta M. CRIS Guidelines (Checklist for Reporting *In-vitro* Studies): a concept note on the need for standardized guidelines for improving quality and transparency in reporting *in-vitro* studies in experimental dental research. J Conserv Dent 2014;17(4):301-4. doi: 10.4103/0972-0707.13633 10.4103/0972-0707.136338 PMC4127685 25125839

[19] 19 - Souza BM, Santi LR, Souza Silva M, Buzalaf MA, Magalhães AC. Effect of an experimental mouth rinse containing NaF and TiF _4_ on tooth erosion and abrasion *in situ* . J Dent. 2018;73:45-9. doi: 10.1016/j.jdent.2018.04.00 10.1016/j.jdent.2018.04.001 29621568

[20] 20 - Wagoner SN, Marshall TA, Qian F, Wefel JS. *In vitro* enamel erosion associated with commercially available original-flavor and sour versions of candies. J Am Dent Ass. 2009;140(7):906-13. doi: 10.14219/jada.archive.2009.0284 10.14219/jada.archive.2009.0284 PMC2730604 19571054

[21] 21 - Comar LP, Gomes MF, Ito N, Salomão PA, Grizzo LT, Magalhães AC. Effect of NaF, SnF(2), and TiF(4) toothpastes on bovine enamel and dentin erosion-abrasion *in vitro* . Int J Dent. 2012;2012:134350. doi: 10.1155/2012/134350 10.1155/2012/134350 PMC3508738 23258978

[22] - Mosquim V, Martines Souza B, Foratori GA Junior, Wang L, Magalhães AC. The abrasive effect of commercial whitening toothpastes on eroded enamel. Am J Dent. 2017;30(3):142-6. 29178759

[23] 23 - Martines de Souza B, Vertuan M, Buzalaf MA, Magalhães AC. The impact of the demineralized organic matrix on the effect of TiF _4_ varnish on the progression of dentin erosive loss. Caries Res. 2017;51(3):264-70. doi: 10.1159/000475534 10.1159/000475534 28521310

[24] 24 - Klimek J, Hellwig E, Ahrens G. Fluoride taken up by plaque, by the underlying enamel and by clean enamel from three fluoride compounds *in vitro* . Caries Res. 1982;16(2):156-61. doi: 10.1159/000260592 10.1159/000260592 6951638

[25] - Azadi-Schossig P, Becker K, Attin T. Chelating effect of citric acid is negligible for development of enamel erosions. Clin Oral Investig. 2016;20(7):1577-87. doi: 10.1007/s00784-015-1634-x 10.1007/s00784-015-1634-x 26572529

[26] - Attin T, Meyer K, Hellwig E, Buchalla W, Lennon AM. Effect of mineral supplements to citric acid on enamel erosion. Arch Oral Biol. 2003;48(11):753-9. doi: 10.1016/s0003-9969(03)00156-0 10.1016/s0003-9969(03)00156-0 14550377

[27] - Shellis RP, Featherstone JD, Lussi A. Understanding the chemistry of dental erosion. Monogr Oral Sci. 2014;25:163-79. doi: 10.1159/00035994 10.1159/000359943 24993265

[28] - Dündar A, Şengün A, Başlak C, Kuş M. Effects of citric acid modified with fluoride, nano-hydroxyapatite and casein on eroded enamel. Arch Oral Biol. 2018;93:177-86. doi: 10.1016/j.archoralbio.2018.06.009 10.1016/j.archoralbio.2018.06.009 29933139

[29] - Richards D. Impact of diet on tooth erosion. Evid Based Dent. 2016;17(2):40. doi: 10.1038/sj.ebd.6401164 10.1038/sj.ebd.6401164 27339233

[30] - Mafla AC, Cerón-Bastidas XA, Munoz-Ceballos ME, Vallejo-Bravo DC, Fajardo-Santacruz MC. Prevalence and extrinsic risk factors for dental erosion in adolescents. J Clin Pediatr Dent. 2017;41(2):102-11. doi: 10.17796/1053-4628-41.2.102 10.17796/1053-4628-41.2.102 28288295

